# The Short-Term Outcome of Seizure and Anti-epileptic Use After Cranial Surgery: A Retrospective Record Review

**DOI:** 10.7759/cureus.33749

**Published:** 2023-01-13

**Authors:** Nada H Abdulaziz, Abeer A Alyami, Alaa A Basuliman, Khlod A Ywsef, Ahlam H Alsulami, Mohammed A Alyousef

**Affiliations:** 1 Department of Medicine, King Abdulaziz University Faculty of Medicine, Jeddah, SAU; 2 Department of Medicine, King Abdulaziz University/King Abdulaziz University Hospital, Jeddah, SAU; 3 Department of Neurological Surgery, King Abdulaziz University Faculty of Medicine, Jeddah, SAU

**Keywords:** outcomes, anti-epileptics, prophylactic, craniotomy, seizure

## Abstract

Objectives

The study aims to correlate craniotomies and their effect on epileptic activity and to assess the impact of prophylaxis anti-epileptic drugs (AEDs) used to prevent seizure activity after craniotomy.

Method

This was a mono-center retrospective review of patients undergoing craniotomy from 2010-2021 at King Abdulaziz University Hospital (KAUH), a tertiary center in Jeddah, Saudi Arabia. The patients were divided into two groups depending on preoperative anti-epileptic drug usage and the occurrence of seizures after the surgery. Out of 192, 24.6% had a seizure before the surgery, while the rest reported no seizure activity. We used descriptive statistics to categorize the study population and applied t-test and chi-square to compare different groups and outcomes.

Results

One-hundred-ninety-two patients were studied: 24.6% had preoperative seizure history and 82.1% were on prophylactic AEDs. The incidence of post-craniotomy seizures was 7.6% in patients with anti-epileptic prophylaxis and 2.7% in those without prophylaxis before the surgery. Almost three-quarters of the patients (72.4%) had surgery for brain tumor resection and redo-craniotomy while the rest (25.5%) were for intracranial hemorrhages (p=0.052). On multivariate analysis, the primary predictor of post-craniotomy seizures was the preoperative history of seizures. Finally, the administration of AEDs does not prevent seizure occurrence after craniotomy (p=0.153). Moreover, the type of prophylaxis and reason for the surgery played no significant role in seizure occurrence.

Conclusion

Post-craniotomy seizures were common, and preoperative AEDs for primary seizure prevention were not associated with a lower incidence of seizures after craniotomy.

## Introduction

Cranial surgery, also known as "craniotomy," is a neurosurgical procedure designed to remove a segment of the skull bone to expose and access the brain, where various diagnostic and therapeutic operations are performed [1]. There are numerous indications for which a craniotomy is performed such as brain tumors, aneurysms, arterio-venous malformations, and epilepsy [2]. As in any surgical intervention, craniotomy can result in detrimental complications; seizures influence 1%-12% of patients [3].

A seizure is defined as an uncontrolled electrical disturbance in the brain that occurs suddenly. It can change an individual's behavior, feelings, and level of consciousness [4]. Seizures after craniotomy can cause serious complications: sensorimotor deficits, ischemic stroke, symptomatic intracranial hemorrhage, hydrocephalus, and redo-craniotomy [5]. Post-craniotomy seizures are proportionally frequent; according to a systemic review in the UK in 2018, the highest incidence of postoperative seizure "occurs within the first month after cranial surgery," and 75% of patients who progress to epilepsy do so within one year of surgery [6-7].

Furthermore, Greenhalgh et al. declared that for non-traumatic brain pathologies, the incidence of seizure post supratentorial craniotomy had been estimated to be around 15%-20%. Thus, the administration of antiepileptic drugs (AED) has been sought to prevent or reduce the occurrence of seizures postoperatively [6]. Moreover, a study formulated in King Abdulaziz Medical Centre, Riyadh, Saudi Arabia, established that from the 32 patients on prophylactic antiepileptic medication, only one (3.1%) experienced a postoperative seizure in comparison to the 12% incidence of post-craniotomy seizures in 92 patients who did not receive ADs prophylaxis [8].

Several studies show that the best current evidence does not provide a well-founded conclusion on whether the use of AED as a prophylactic treatment is effective in preventing the occurrence of seizures. A systemic review done in 2020 found that out of 10 randomized controlled trials (RCTs), only "two trials reported a statistically significant advantage for AED treatment compared to controls for early seizure occurrence;" there were no well-defined or statistically significant variances between AEDs and control treatment in the other studies [6]. Furthermore, AEDs are linked with significant adverse events [9].

Thus, our aim in this research is to measure the impact and effectiveness of AED prophylactic usage to prevent seizure incidence post-craniotomy as well as associating surgeries and their effect on seizure activity seen in (King Abdulaziz University Hospital, Jeddah, Saudi Arabia) patients who have undergone craniotomy from 2010-2021.

## Materials and methods

Patients and setting

This study was a retrospective record review conducted in June 2021 of all patients admitted to King Abdulaziz University Hospital (KAUH), a tertiary medical center in Jeddah, Saudi Arabia, department of neurosurgery, and approved by the research ethics committee of KAUH. Patients were reviewed from the Phoenix, the KAUH records database, and a convenience sample was conducted of all patients who underwent craniotomy in King Abdulaziz University Hospital that fit the inclusion criteria.

Inclusion criteria were adult patients aged 18 years and above undergoing craniotomy between 01/01/2010 and 31/06/2021. Patients who had seizures before the surgery, and improved, worsened, or remained static post-op. Patients who developed seizures post-op and reported no seizure activity before. Patients with at least six months of follow-up after the surgery. Exclusion criteria were patients lost to follow-up; patients with less than six months follow-up after the surgery; patients who developed the following intracranial complications due to the surgery: hemorrhage, infection, or stroke; patients with a previous history of meningitis, ventriculitis, or encephalitis; patients with a history of epilepsy during childhood; and death within six months of follow-up.

The primary outcome measure was the proportion of postoperative seizures (categorized into focal, generalized, and overall seizure rate) during the study follow-up time. Secondary outcome measures included hospitalization time and recurrent seizures. Seizures were defined as clinically diagnosed seizures, where AED treatment was commenced since routine postoperative electroencephalography (EEG) was not done in our institutions. Early seizures occurring during hospitalization were reported by the treating physician and documented in the patient's patient's medical records. Late-occurring seizures were usually reported by the patients or their families to the treating general physician or the treating emergency room physician, who then referred the patients to our care.

Statistical analysis

Microsoft Excel 2021 (Microsoft Corporation, Redmond, WA) was used for data entry, and the Statistical Package for Social Science (SPSS) version 21 (IBM Corp. Armonk, NY) was used for statistical analysis. Continuous variables were presented as means with standard deviations and categorical variables as frequencies with percentages.

To assess the risk factors of post-craniotomy seizures, a multivariate analysis was performed with the following variables entered into the model: preoperative antiepileptic drug therapy, the reason for surgery, laboratory variables such as blood glucose (mmol/L), blood urea nitrogen (BUN) (mmol/L), creatinine (µmol/L), sodium (mmol/L), calcium (mmol/L), magnesium (mmol/L), and phosphorous (mmol/L). The chi-square test was used to assess differences between categorical variables. The t-test was performed to evaluate differences between continuous variables. The results were presented as odds ratios (ORs) with 95% confidence intervals (CIs). A test with a P-value < 0.05 was considered significant.

## Results

Patients’ characteristics

In the past 10 years (2010-2021), 192 patients had craniotomy at KAUH for various causes, with a mean age of 47.6±18.6 years, and were predominantly (52.6%) males. Younger patients (44.2±17.8) were more likely to receive preoperative AEDs (p=0.001), as Table 1 shows.

**Table 1:** Characteristics of patients according to the incidence of seizures after craniotomy Equality of variance < 0.05, †equality of variance < 0.05, p-value of the Mann-Whitney Test) non-parametric(=0.08) *4 missing data

Variables	All Participants (n=192)	Preoperative antiepileptic drugs (n=118)	No Preoperative antiepileptic drugs (n=74)	P-value
Age (years), mean±SD	47.6±18.6	44.2±17.8	53.2±1	0.001
Male, n (%)	101 (52.6)	65 (64)	36 (36)	0.385
Comorbid conditions, yes n (%)	72 (37.5)	40 (33.9)	32 (43)	0.193
Hypertension (HTN)	68 (35.4)	44 (64.7)	24 (35.3)	0.494
Diabetes Milletus (DM)	49 (25.5)	30 (61.2)	19 (38.9)	0.969
Coronary Artery Disease (CAD)	4 (2.1)	2 (50)	2 (50)	0.634
Cardiovascular Disease (CVD)	11 (5.7)	8 (72.7)	3 (27.3)	0.429
Epilepsy	28 (24.6)	23 (82.1)	5 (17.9)	0.015
Other	55 (28.6)	35 (63.6)	20 (36.3)	0.154
Reason of surgery*, n (%)				
Tumor resection & Redo-craniotomy	139 (72.4)	90 (64.8)	49 (35.0)	0.052
Intracranial Hemorrhage	49 (25.5)	24 (48.9)	25 (51.0)	0.052
Laboratory data day 1 post-operative, n (mean±SD)				
Blood Glucose (mmol/L)	147 (8.8±3.2)	95 (9.1±3.4)	52 (8.3±2.7)	0.162
Blood Urea Nitrogen (BUN) (mmol/L)	187 (4.92±3.2)	115 (4.8±3.0)	72 (5.1±3.5)	0.636
Creatinine (µmol/L)	187 (73.1±35.8)	115 (69.8±30.4)	72 (78.5±42.6)	0.108
Sodium (mmol/L)	188 (139.4±5.4)	116 (139.8±4.6)	72 (138.8±6.5)	0.254
Calcium (mmol/L)	157 (2.1±0.2)	101 (2.1±0.2)	56 (2.1±0.2)	0.502
Magnesium (mmol/L)	158 (0.8±0.1)	102 (0.8±0.2)	56 (0.8±0.1)	0.845
Phosphorous (mmol/L)	155 (1.2±0.3)	100 (1.2±0.3)	55 (1.2±0.3)	0.696
Length of stay at Hospital (days), mean±SD	19.3±20.8	21.0±22.1	16.6±18.3	0.154
Discharge on AED, (yes, n, %)	121 (63.0)	107 (90.9)	14 (18.9)	0.000
Follow-up Seizures, n(%)	23 (12)	18 (15.3)	5 (6.8)	0.078

Preoperative seizures were generalized in 17.8% and partial in 15.2%. People with a history of seizures are more likely to take prophylaxis (p=0.015) and be discharged on antiepileptic drugs (63%, p=0.000). Patients selected in this study had a follow-up period of at least six months. Out of these patients, only 12% had this period of follow-up documented. However, prophylaxis does not significantly correlate with whether they will get follow-up seizures.

Out of 192, 52.6% (101) were males while the rest were females, though the gender did not correlate with the incidence of seizures (p=0.35). The indications for surgery varied; the majority was for tumor resection and redo craniotomies (72.4%), in which 64.8% received AEDs preoperatively and for intracranial hemorrhage, 25.5%, with 48.9% having received AEDs preoperatively. However, there is no significant correlation between the reason for craniotomy and seizure occurrence (p=0.052). However, there were four missing data for the indication of surgery. Twenty-eight (24.6%) patients had a seizure history before craniotomy, and most of those patients (82.1%) were on antiepileptic drug prophylaxis, as shown in Table 1.

Preoperative seizures were generalized in 17.8% and partial in 15.2% of the patients. People with a history of seizures are more likely to take prophylaxis (p=0.015) and be discharged on antiepileptic drugs (63%, p=0.000), as depicted in Table 2. Patients selected in this study had a follow-up period of at least six months. Out of these patients, only 12% had this period of follow-up documented. However, prophylaxis does not significantly correlate with whether they will get follow-up seizures.

**Table 2:** Effect of drug prophylaxis on seizure status in craniotomy patients *No AED: No anti-epileptic drugs **AED: anti-epileptic drugs

Effect of drug prophylaxis on seizure status in craniotomy patients							
	Preoperative Seizure occurrence (42)				Postoperative Seizure occurrence (11)		
	No Seizures	Generalized	Focal	P-value	No Seizures	Seizures	P-value
No AED* (74), n (%)	71 (95.9)	1 (1.4)	2 (2.7)	0.000	72 (97.2)	2 (2.7)	0.153
AED** (118), n (%)	79 (66.9)	21 (17.8)	18 (15.2)		109 (92.4)	9 (7.6)	
Phenytoin (41), n (%)	33 (80.4)	5 (12.1)	3 (7.3)	0.762	37 (90.2)	4 (9.8)	0.211
Levetiracetam (81), n (%)	60 (74.0)	11 (13.6)	10 (12.3)	0.510	75 (92.6)	6 (7.4)	0.393
Phenytoin & Levetiracetam (96), n (%)	75 (78.1)	14 (14.6)	7 (7.3)	0.297	90 (93.8)	6 (6.2)	0.225
Other(10), n (%)	5 (50.0)	2 (20.0)	3 (30.0)	0.060	10 (100)	0 (0)	0.423

## Discussion

Our study aimed to analyze the use of AED prophylaxis and its effect on post-craniotomy seizure occurrence. Our study's main findings proved that people with a history of seizures were more commonly given AED prophylaxis (p=0.015)and discharged with it as secondary prophylaxis (p=0.000). Moreover, younger age (44.2±17.8) was significantly correlated with administering AEDs as prophylaxis (p=0.001). On the other hand, there was no significance between AED prophylaxis and seizure occurrence. Other factors that we analyzed included gender, indication for surgery, and type of AED administered were also non-significant to seizure occurrence post-operatively.

Previous literature states that patients with a history of preoperative seizures were more likely to experience postoperative seizures [5]. In our research, patients with a prior history of seizures were significantly given AED and even discharged with it as secondary prophylaxis. We established that there is no significant correlation between AED prophylaxis and seizure occurrence post craniotomy, neither did the indication of the surgery show any significance, which was proven in a systematic review of six randomized trials on 1398 patients who had craniotomies for different causes found insufficient evidence of the effectiveness of AEDs as prophylaxis to prevent post-craniotomies seizure [6]. Similarly, the type of prophylaxis played no significant role in seizure occurrence; a meta-analysis of five trials analyzed three AEDs (phenobarbital, phenytoin, and valproic acid) that did not affect seizure prevention at one week [10].

On the contrary, a phase II trial compared levetiracetam with phenytoin and found a lower incidence of early post-craniotomy seizures 1.4% versus 15.1% (p=0.005) and a lower rate of side effects in the levetiracetam group [11].

The findings of this study should be interpreted in light of its strengths and limitations. This study includes a relatively high number of patients with primary brain tumors of different histology undergoing surgical resections and reflects general neurosurgery practice. Due to its retrospective nature, it was impossible to control or account for various factors, including preoperative seizure characteristics, seizure treatment, tumor depth, site, and surgical details. On the other hand, the study was conducted at a single center, resulting in a limited sample size, which may have affected the insignificance of the outcomes. A clinical trial may be helpful in such a case.

## Conclusions

In conclusion, our results have shown that preoperative AEDs for primary seizure prevention were not associated with a lower incidence of seizures after craniotomy, which proves the null hypothesis. Our study's strengths and flaws affected how we interpreted our results. Some of the limitations we encountered were the study's retrospective nature and the lack of comprehensive information about the surgery, relying on history taking only (subjective evidence) without any objective tools (such as EEG) to assess if the patient truly had a seizure was one of our limitations. Comparatively, our single-setting study had fewer participants than prior single-setting studies. Though this topic remains immensely debatable, and further studies, such as random clinical trials, are required to assess the clinical effectiveness of prophylactic AED on seizure occurrence after a craniotomy to reach a conclusive result.
